# Breastfeeding in patients with peripartum cardiomyopathy: clinical outcomes and physician counseling

**DOI:** 10.1186/s13006-024-00673-6

**Published:** 2024-10-29

**Authors:** Angelina Noll, Kris R. Kawamoto, Maya T. Dassanayake, Laura Leuenberger, Stephanie M. Spehar, Jenny Wu, Elizabeth Langen, Melinda B. Davis

**Affiliations:** 1 Department of Medicine, University of Michigan Medical School, Ann Arbor, MI USA; 2 Cardiovascular Diseases, Queen’s University Medical Group, Honolulu, HI USA; 3 Department of Medicine, Division of Cardiology, University of South Carolina-Greenville, Greenville, SC USA; 4 Department of Medicine, Division of Pulmonary, Critical Care and Sleep, Ohio State University, Columbus, OH USA; 5 Department of Medicine, Division of Cardiovascular Medicine, Department of Obstetrics and Gynecology, University of Michigan, Ann Arbor, MI USA; 6 Department of Obstetrics and Gynecology, Division of Maternal Fetal Medicine, University of Michigan, 1500 E. Medical Center Drive, Ann Arbor, MI 48105 USA

**Keywords:** Peripartum cardiomyopathy, Postpartum cardiomyopathy, Pregnancy-associated heart failure, Breastfeeding, Lactation, Myocardial recovery

## Abstract

**Background:**

Peripartum cardiomyopathy (PPCM) is a form of heart failure occurring towards the end of pregnancy or in the months following delivery. Concerns regarding the role of prolactin (the polypeptide hormone responsible for lactation) driving the pathogenesis of PPCM have led experts to discourage patients from breastfeeding; however, limited clinical data exist. We sought to (1) determine whether lactation was associated with less cardiac recovery and (2) assess the counseling about breastfeeding given to patients at the time of their initial diagnosis.

**Methods:**

Patients diagnosed with PPCM from 1999 to 2019 were identified through detailed chart review and demographic characteristics, comorbidities, outcomes, and lactation status were collected. Cardiac recovery was defined as left ventricular ejection fraction (LVEF) 55% or higher. A survey about breastfeeding and patient experience was administered by mail. Patients were only included in this analysis if definitive information about lactation status was documented.

**Results:**

Of 220 patients with confirmed PPCM, lactation status was known definitively in 54 patients; of these, 18 (33%) had breastfed for at least 6 weeks and 36 (67%) did not breastfeed. There were no significant differences in the breastfeeding and non-breastfeeding groups related to baseline LVEF, age, race, gestational diabetes, smoking, hypertensive disorders of pregnancy, and medication treatments. Despite similar baseline LVEF at the time of diagnosis, there was no statistically significant difference in cardiac recovery based on lactation status. In a subset of patients with severe cardiac dysfunction at the time of diagnosis, there remained no significant differences in recovery based on lactation status. Of the 34 survey respondents, 62% were told not to breastfeed due to their diagnosis or concerns regarding safety of medications, and none were encouraged to breastfeed.

**Conclusion:**

In this retrospective cohort, lactation was not associated with lower rates of myocardial recovery. Importantly, a majority of patients had received counseling that they should not breastfeed. Future studies of the role of lactation in PPCM are needed in order to better understand the impact of breastfeeding and improve patient counseling.

## Background

Peripartum cardiomyopathy (PPCM) is an idiopathic form of heart failure with reduced left ventricular systolic function presenting towards the end of pregnancy or in the months following delivery [1]. Outcomes range from full myocardial recovery to heart transplantation and death. At the time of diagnosis, patients are frequently instructed to stop breastfeeding. This recommendation is largely based on concerns surrounding the role of the hormone prolactin driving the pathogenesis of PPCM [2, 3].

Few prior studies of PPCM and lactation exist [4]. In this study, we build upon these prior studies by analyzing a cohort of well-characterized PPCM patients with known lactation status to determine a correlation with cardiac recovery. Additionally, a survey was administered to elicit patient experiences related to breastfeeding.

## Methods

Patients who had received a portion of their care at University of Michigan, a quaternary care center with high numbers of heart failure referrals, and had been diagnosed with PPCM from 1999 to 2019 were identified through chart review using international classification of diagnosis (ICD) codes and free text search engines [5]. Patients were included even if their initial diagnosis and/or treatment plan occurred at another center, provided that sufficient follow-up information at our center was available for analysis. Charts were thoroughly reviewed to ensure all patients met the formal diagnostic criteria [6]. Data regarding demographic characteristics, comorbidities, outcomes, and breastfeeding were collected. A survey about lactation status and patient experiences from the time of their initial diagnosis was administered first in 2017 and then to an additional cohort in 2020 after additional patients were identified, in to increase our sample size. Patients were asked if they had breastfed (or expressed breastmilk) and for what duration. The survey included a question asking what information they had received at the time of diagnosis; the possible responses were as follows: (A) told NOT to breastfeed due to diagnosis; (B) told NOT to breastfeed due to medications; (C) told I SHOULD breastfeed; (D) Not given any information; (E) Do not remember.

PPCM recovery was defined as left ventricular ejection fraction (LVEF) *≥* 55%. The lactation cohort was defined as breastfeeding/pumping for 6 weeks or longer. Additionally, a subset of patients with severe cardiac dysfunction at the time of diagnosis (LVEF of 30% or lower at baseline) was also analyzed. Statistical comparisons were conducted using chi square and Fischer Exact analyses, and p-value < 0.05 was considered statistically significant. This study was approved by our Institutional Review Board.

## Results

**Fig. 1:**
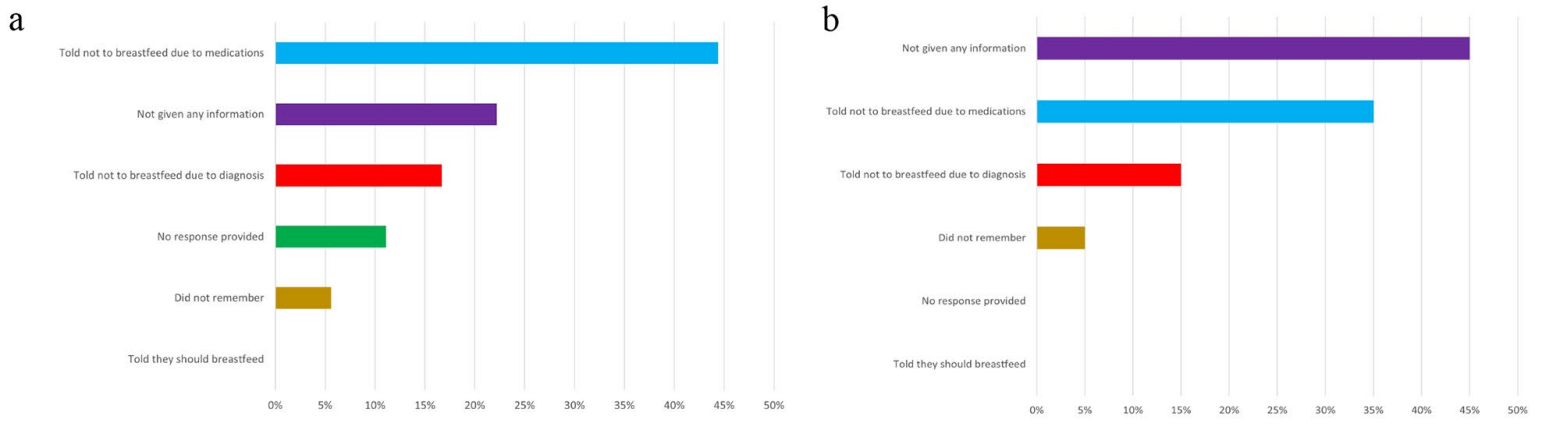
Lactation survey responses regarding breastfeeding demonstrate large gaps in information and counseling: (**A**) Non-breastfeeding respondents; (**B**) Breastfeeding respondents

Of 220 patients with confirmed PPCM, breastfeeding/lactation status and duration was known definitively in 54 patients; of these, 18 (33%) breastfed/pumped and 36 (67%) did not breastfeed. There were no significant differences in the lactation and non-lactation groups related to baseline LVEF, age, race, gestational diabetes, smoking, and hypertensive disorders of pregnancy (Table 1). Medication treatments were known for 48 of the 54 patients and there were no significant differences between the groups. Bromocriptine was used in 4 patients in the non-lactation group. Four of 18 (22%) recovered in the lactation group compared with 13 of 36 (36%) in the non-lactation group (*p* = 0.48). Among a subgroup of patients with severe cardiac dysfunction with initial LVEF ≤ 30% (*n* = 21), there remained no significant difference in recovery status between the lactation group (2 of 5, 40%) and the non-lactation group (8 of 16, 50%) (*p* = 0.70). Of the 34 survey respondents, 62% were told not to breastfeed due to their diagnosis or medications and none were encouraged to breastfeed (Fig. 1).

**Table 1:** Demographic and clinical characteristics of women with peripartum cardiomyopathy and cardiac outcomes

	No Lactation	Lactation	*p* value (*p* < *0.05)*
	(n = 36)	(n = 18)	
Age at diagnosis (yrs)			
Mean *±* SD	31 *±* 6	32 *±* 6	0.56
African American Ancestry, n (%)	8 (22)	3 (17)	0.73
Hypertension (pooled), n (%)	21 (58)	5 (28)	0.12
Pre-Existing Hypertension, n (%)	7 (19)	3 (17)	1.0
Gestational Hypertension, n (%)	8 (22)	1 (6)	0.25
Preeclampsia, n (%)	17 (47)	4 (22)	0.21
Gestational diabetes, n (%)	2 (6)	2 (11)	0.57
Smoking, n (%)	9 (25)	4 (22)	1.0
ACEi/ARB, n (%)*	34 (94)	14 (78)	1.0
Beta Blocker, n (%)*	32 (88)	14 (78)	1.0
Spironolactone, n (%)*	18 (50)	4 (22)	0.21
Digoxin, n (%)*	7 (19)	5 (28)	0.47
Diuretic, n (%)*†	33 (92)	12 (67)	0.20
EF at diagnosis, mean *±* SD	28 *±* 13	30 *±* 13	0.59
EF at 12 months, mean *±* SD	39 *±* 16	41 *±* 15	0.65
Recovered, n (%)	13 (36)	4 (22)	0.48

ACEi = angiotensin-converting enzyme inhibitor; ARB = angiotensin receptor blocker; EF = ejection fraction; *Medication status known in 48 patients; †Diuretics: furosemide, bumetanide; “Recovered”: defined as EF ≥ 55%

## Discussion

This is one of few studies to focus directly on the impact of lactation in a population with well-defined PPCM. We found no correlation between lactation and adverse outcomes, even among those with baseline LVEF ≤ 30%. Through survey responses, we found patients with PPCM rarely received adequate counseling regarding lactation.

In 2007, Helfiker-Kleiner et al. showed that a degradation product of prolactin (the hormone responsible for lactation) drives the proposed pathogenesis of PPCM in murine models, and inhibiting prolactin release with bromocriptine prevented PPCM in mice [2]. In humans, prolactin levels decline after birth and reach nonpregnant levels by seven days postpartum if not breastfeeding. Plasma prolactin levels increase significantly with lactation and remain elevated while breastfeeding. Thus, experts have hypothesized that the prolonged elevated prolactin levels during breastfeeding may delay or decrease the chances of recovery in women with PPCM. The European Society of Cardiology guidelines recommend consideration of preventing lactation in PPCM due to concerns regarding the role of prolactin and the high metabolic demands of lactation (Class IIb) [3]. For this reason, suppression of prolactin with bromocriptine is standard of care in Europe. However, given the absence of a randomized placebo-controlled trial in North America, the Randomized Evaluation of Bromocriptine in Myocardial Recovery Therapy (REBIRTH) study is currently sponsored by the National Heart, Lung, and Blood Institute [7] and is actively enrolling at sites across North America [8]. Patients will be randomized to bromocriptine versus placebo, along with an observational-only cohort for patients who breastfeed.

Our patient survey revealed a high percentage were instructed to avoid breastfeeding due to medications. However, the American Academy of Pediatrics, National Institutes of Health, and other experts have indicated that multiple heart failure medications (i.e. metoprolol, carvedilol, captopril, enalapril, benazepril, digoxin, spironolactone) can be used safely with lactation [1, 9–12]. During the study timeframe, the use of angiotensin receptor/neprilysin inhibitors and sodium-glucose transport protein 2 inhibitors had not yet become standard of care, for which lactation safety data remains insufficient.

Our study is one of few to directly address the impact of lactation in the setting of PPCM. A strength of this study was the fact that our detailed chart review ensured all patients met strict diagnostic and inclusion criteria, rather than reliance on administrative codes. Limitations of our study include the fact that our survey tool was not previously validated. In addition, the long duration of follow-up may contribute to recall bias in the survey responses.

## Conclusions

In this retrospective cohort, lactation was not associated with a reduction in myocardial recovery. Many patients were either instructed not to breastfeed or were not given any information about lactation. Future studies, such as the ongoing REBIRTH clinical trial, can add to our understanding of the role of lactation in PPCM.

## Data Availability

The datasets generated during the current study are not publicly available due to the desire to protect patient confidentiality in this small cohort study but de-identified data may be made available from the corresponding author on reasonable request and with institutional permission regarding data sharing.

## Abbreviations

ICD: International classification of diseases
LVEF: Left ventricular ejection fraction
PPCM: Peripartum cardiomyopathy
REBIRTH: Randomized Evaluation of Bromocriptine in Myocardial Recovery Therapy
